# Digital Overuse and Addictive Traits and Their Relationship With Mental Well-Being and Socio-Demographic Factors: A National Population Survey for Wales

**DOI:** 10.3389/fpubh.2021.585715

**Published:** 2021-06-16

**Authors:** Mark A. Bellis, Catherine A. Sharp, Karen Hughes, Alisha R. Davies

**Affiliations:** ^1^Public Health Collaborating Unit, School of Health Sciences, College of Human Sciences, Bangor University, Wrexham, United Kingdom; ^2^Policy and International Health, WHO Collaborating Centre on Investment for Health and Well-being, Public Health Wales, Wrexham, United Kingdom; ^3^Research and Evaluation Division, Knowledge Directorate, Public Health Wales, Cardiff, United Kingdom

**Keywords:** digital overuse, well-being, mental health, public health, digital technology, socio-demographics

## Abstract

**Introduction:** Population health concerns have been raised about negative impacts from overuse of digital technologies. We examine patterns of online activity predictive of Digital Overuse and Addictive Traits (DOAT). We explore associations between DOAT and mental well-being and analyse how both relate to self-reported changes in self-esteem, perceived isolation, and anxiety about health when individuals use the internet for health purposes.

**Methods:** A cross-sectional nationally representative household survey of adults using stratified random sampling (compliance 75.4%, *n* = 1,252). DOAT was measured using self-reported questions adapted from a social media addiction scale (failure to cut down use, restlessness when not using, and impact on job/studies and home/social life in the last year), combined into a single DOAT score. Higher DOAT score was defined as >1 standard deviation above population mean. The Short Warwick-Edinburgh Mental Well-being Scale was used to measure mental well-being. Analyses were limited to those with internet access (*n* = 1,003).

**Results:** Negative impacts of digital technology use on work and home/social lives were reported by 7.4% of respondents. 21.2% had tried but failed to cut down use in the past year. Higher DOAT was associated with higher social media and internet use but also independently associated with greater risks of low mental well-being. Higher DOAT was associated with both improvement and worsening of self-esteem, perceived isolation and anxiety about health when using the internet for health reasons, with no change in these outcomes most likely in those with lower DOAT. Lower mental well-being was associated with a similar bi-directional impact on perceived isolation and was also associated with worsening self-esteem.

**Conclusions:** Substantial proportions of individuals report negative impacts on home, social and working lives from digital technology use, with many trying but failing to cut down use. Individuals with higher DOAT may experience improvements or worsening in self-esteem and other measures of mental well-being when using the internet for health purposes. From a public health perspective, a greater understanding of risk factors for digital overuse, its impacts on well-being and how to reasonably limit use of technology are critical for a successful digital revolution.

## Introduction

The establishment of digital technologies as a central component of home and work environments has been accompanied by debate over their impact on health and well-being, including what constitutes reasonable levels of use [e.g., time on the internet (1)]. More than half of the world's population now uses the internet with continued growth annually (2) across both high income and increasingly low and middle-income countries (3). Specifically, smartphone use has increased dramatically. For example, in the UK, over the course of a decade (2008–18) smartphone use in those aged 16+ years increased from 17% to 78% (4), while in Indonesia, over 5 years (2013–18) ownership increased from 11% (5) to 42% (3). While the benefits of digital technologies in facilitating social contact and access to health information have been realised through the COVID-19 pandemic (6), this is likely to have further increased use of and reliance on digital technologies as physical contact is switched to virtual contact; data is already emerging that populations have used more social media compared to pre-pandemic times (7). However, an escalating presence and reliance on digital technology has developed in the absence of robust evidence on what constitutes an appropriate or harmful level of use. Consequently, health concerns have been raised about the potential negative impacts that overuse of technology may have broadly on population mental well-being (8, 9), as well as on individuals whose health and social circumstances leave them especially vulnerable (10, 11).

For some time, excessive use of digital technology has been described from an addiction perspective or with a focus on extreme behaviours (12), with the term “technology addiction” often applied to more extreme compulsion and dependence (8). More recently, however, there has been an increased focus on the wider population phenomenon and public health implications of perceived digital overuse [PDO; the difference between the extent of practised and desired internet usage (9, 13)]. Although not necessarily associated with more extreme compulsion and dependence (8), overuse can have negative impacts on other aspects of life (9) and may affect large proportions of the population. For instance, a 2018 British survey found 43% of adults agreed that they *spend too much time online* (4). Thus, whilst the health impacts of technology addiction may affect a small proportion of people, wider PDO may be relevant to a much greater proportion (13). Overall, studies suggest there can be both health benefits and negative health consequences from using digital technologies (14). Relationships are compounded not only by levels of use (15, 16) but also by the purpose of use and content being viewed (17, 18). Thus, individuals may be using online websites, social media and other digital platforms to inform and address a wide range of personal well-being issues [e.g., accessing information about general health, self-diagnosing a health condition, and finding emotional support online (19)].

With a focus on public health and digital technology still emergent, there is currently limited information available on the balance between health risks and benefits (20). Even less is known on how these vary across different populations or are impacted by health issues, with evidence on impacts such as poor mental well-being in its infancy (21). Nevertheless, governmental guidance on how digital technology can be used safely is beginning to appear, albeit currently only for younger populations and drawing from a limited evidence base (22–25). However, unlike some public health interventions where the long-term goal is to eliminate a behaviour (e.g., smoking), the goal with digital technologies is more complex and involves understanding the reasons for use and the potential benefits it can impart as well as how it potentially detracts from other health-imparting activities and face to face non-digital interactions (26). To date, studies of both technology addiction and PDO have found associations between digital use and lower well-being; although impacts may be smaller in the case of PDO (9, 13). Regardless, with neuroscience informing even more appealing and immersive interfaces (27), reliance on and use of technology is predicted to continue to increase (28) and consequently, it is critical that the potential risks to public health are better understood.

Much of the research exploring the broader impact of digital technology on health has focused on adolescents (20, 29–34). Less attention has been given to other adult demographics leaving major gaps in understanding, especially in older age groups. For adolescents, negative associations have been found between technology use and mental health including depression, social isolation and cyberbullying (34). However, moderate use can be advantageous (15). Positive associations have been found with online activities including increased self-esteem and perceived social support (34) and decreased psychological distress (35). Further, even in the better-studied younger populations, debate continues as to the strength of reported negative impacts of technology on health and whether they constitute sufficient concern to justify stronger public guidelines (36).

Predictions of continued escalations in both the numbers of individuals using digital technologies and the amount of digital time each individual consumes (28) make it critical that we better understand who is at risk of digital overuse and addiction, and how these relate to other vulnerabilities such as low mental well-being. Here we use a national survey to measure, across socio-demographic groups, the prevalence of negative impacts of digital technology use on home, social and work life and on failure to cut down or restlessness when stopping use. We combine these measures into a single overuse and addiction scale and examine its internal consistency. We test the hypothesis that current mental well-being is associated with higher overuse and addiction scores and examine if such relationships are affected by other factors (e.g., levels of online activity or other common addictive behaviours). Finally, we test the hypothesis that self-reported changes in dimensions of mental well-being resulting from internet use (self-esteem, perceived isolation, and anxiety about health) are predicted by scoring higher on the scale. As this study was exploratory and current evidence is conflicting, no directional hypotheses are presented.

## Methods

A cross-sectional nationally representative household survey was undertaken in Wales between April and June 2018. With no existing data on the prevalence in the national population of the variables comprising the DOAT scale, we adopted a target sample size of 1,250 interviews. Sample size was based on previous national surveys also undertaken to provide adequate samples to capture key socio-demographic groups in Wales (37, 38). In order to ensure the sample was nationally representative by age, gender, deprivation level, and rurality, a stratified random probability sampling framework was employed. Stratification was undertaken by Health Board area (*n* = 7) to ensure national coverage. Within each Health Board, Lower Super Output Area (LSOAs; geographic areas with a population mean of ~1,500 people) were categorised into rural/urban classifications (39) and into deprivation quintiles using the Welsh Index of Multiple Deprivation [WIMD; (40)]. The WIMD is a composite measure of multiple factors combined on a small geographical footprint. LSOAs were then randomly selected (*n* = 125) in proportion to the totals by deprivation quintile and rurality within each Health Board. To avoid selection bias, households were selected at random within selected LSOAs using the postcode address file of all households in each sampled LSOA. Each selected household (*n* = 3,870) was sent a letter outlining the reasons for the study and providing household recipients the opportunity to opt out by phone or email; 253 opted out from participating at this stage.

Households remaining in the study were visited by trained interviewers for a face-to-face interview conducted using computer-assisted personal interviewing (CAPI) technology. Interviews were undertaken on all days of the week between 9 a.m. and 8 p.m. Each residence was visited a maximum of five times to make contact with residents and establish if they accepted or refused participation. Thus, once contact was made the interviewer provided a letter of authority outlining the purpose of the study and its anonymous and confidential nature. The interviewer also informed individuals again that they could withdraw from the study. To further reduce selection bias, only a single resident from each household could participate, and where more than one resident was eligible for inclusion, surveyors asked to speak to the resident with the next birthday. Study inclusion criteria were resident in Wales, aged 16+ years, and cognitively able to participate (i.e., interviewer assessed the resident was capable of understanding the questions). Questions were offered in English and Welsh with other languages as required (Urdu 3, Romanian 1, Bengali 1). To achieve the desired sample target, contact was made with 2,480 households, of which 31 did not meet the study inclusion criteria (e.g., business property, no Welsh resident). Of the 2,449 remaining, 789 were deemed vacant, non-responsive or residents were otherwise unavailable at the time of contact. Of the remaining 1,660, 408 withdrew at the doorstep, resulting in a final sample of 1,252 and compliance at the door of 75.4% (1,252/1,660). Although initial inclusion criteria were 16+ years, there were difficulties accessing 16–17-year-olds. Therefore, for analysis of digital technology use here, only those individuals aged 18+ who had internet access at their place of residence or identified that they accessed the internet at least weekly elsewhere were included (*n* = 1,064; 85.0% of sample). Another 61 were excluded as they did not provide complete demographic data or answer all questions relevant to this analysis (final sample: *n* = 1,003).

Respondents were informed that the term “internet” would be used to include a range of activities such as “going online, accessing websites, using social media and looking for information.” They were also told that “technology” would be used to include devices to connect to the internet such as mobile phones, computers, and tablets. Here such devices are referred to as digital technologies. To measure different aspects of individuals' Digital Overuse and Addictive Traits (DOAT), we used three questions from the Bergen Facebook Addiction Scale [FAS (41); Appendix Table 1 in Supplementary Material for full questions and response option for each key dependent and independent variable] and switched the focus from Facebook to broader digital technology, with one question replicated to assess an additional context (home and social life). Thus, the questions asked how often in the past year, had they: tried but failed to cut down their use of technology; used technology so much that it has had a negative impact on their job/studies or their home and social life; and become restless or troubled when not allowed to use it. Other addiction scales were considered (42) but the adapted FAS questions selected due to their generic nature and simple question wording. For each question, respondents answered using a Likert scale (never 1, rarely 2, sometimes 3, often 4, and very often 5). In order to create categories of adequate size for analysis, scores were dichotomised into “no” (never or rarely) and “yes” (sometimes; often; very often). A “not applicable” response was available for impacts on work question for those not in work or studying (*n* = 61). Answers were also summed into a DOAT Scale (range: 1–20; Appendix Table 1 in Supplementary Material for full question details). For those not in work or studying, the DOAT score used the sum of the scores from the three other questions (failed to cut down, restless when stopped using, negative impact on home/social) plus their mean. The scale showed acceptable internal consistency (Cronbach's alpha: 0.734). Consistent with our treatment of mental well-being scales (see later in Methods), we used a standard statistical approach (43) to create a category of higher DOAT score, with individuals with a DOAT score greater than one SD above the sample mean (mean = 5.7, SD = 2.5) categorised as having a higher DOAT.

Social media use was measured across seven different platforms (Twitter, Facebook, LinkedIn, Instagram, YouTube, WhatsApp, Snapchat) with individuals self-classifying use of each of the platforms on a Likert scale from “never” to “several times a day” (Appendix Table 1 in Supplementary Material). For each platform, individuals were allocated a score: 0, never; 1, weekly or less; 2, daily; 3, several times a day. Scores for each individual were summed and people were then classified into low (≤3), moderate (4–7) and high (≥8) tertiles of social media use. Frequency of internet access through different digital technologies was treated similarly. Thus, frequency of phone, computer, laptop, and desktop and tablet access to the internet were each rated (0, never; 1, weekly or less; 2, daily; 3, several times a day; Appendix Table 1 in Supplementary Material) and a composite total measure was created and people again classified into low, moderate and high tertiles. Finally, respondents were asked whether their experience of using the internet and technology to support their health had beneficial, detrimental or no impact on their well-being across three measures (self-esteem, feeling isolated, and anxiety about health; Appendix Table 1 in Supplementary Material).

Respondents were asked to self-report their mental well-being using the Short Warwick Edinburgh Mental Well-being Scale (SWEMWEBS, see Appendix Table 1 in Supplementary Material) and a composite metric score was derived in line with the scale guidance (43). Consistent with other studies (43, 44), lower mental well-being (LMWB) was defined as one standard deviation or more below the sample mean (SWEMWEBS: mean = 24.4, SD = 4.5). Other potentially addictive behaviours (smoking and alcohol use) were analysed as binary variables; current smoker (yes/no; current daily tobacco smoker = yes) and current binge drinker (yes/no; i.e., an individual who consumes 6 or more alcohol drinks in a single drinking occasion on a weekly or more frequent basis; Appendix Table 1 in Supplementary Material for full questions and definitions). Socio-demographic information was collected for age categories (Table 1), gender, ethnicity [self-defined using UK census categories (45) and for the purpose of analysis categorised into white British and other due to small numbers in individual non-white groups] and rurality [derived from (39) using seven categories and dichotomised into urban and rural].

**Table 1:** Demographic distribution of Digital Overuse and Addiction Traits (DOAT).

			**% Categorised “yes”^#^**				**% Higher DOAT^∧^**
		***n***	**Failed to cut down**	**Restless when stopped using**	**Negative impact on home/social**	Negative impact on work^*^	
	All	1,003	21.2	12.5	7.4	7.4	13.6
Age (years)	18–29	180	31.7	23.9	12.2	13.6	22.8
	30–39	213	30.5	15.0	13.6	9.2	20.7
	40–49	180	28.3	12.2	8.9	9.6	15.6
	50–59	168	13.7	7.7	1.8	3.1	7.1
	60+	262	6.5	6.1	1.5	2.3	4.2
	*X^2^*		67.882	35.716	39.723	24.790	48.307
	P		<0.001	<0.001	<0.001	<0.001	<0.001
Deprivation	Poor 1	177	24.9	18.6	10.7	9.0	20.9
	2	203	21.7	9.9	5.9	5.3	10.3
	3	188	19.6	11.1	5.8	7.3	11.7
	4	213	20.7	12.2	8.0	8.0	14.1
	Affluent 5	222	19.8	11.7	6.8	7.7	11.7
	*X^2^*		1.993	7.820	4.437	2.002	11.186
	P		0.737	0.098	0.350	0.735	0.025
Gender	Male	464	18.5	10.3	6.7	9.0	12.9
	Female	539	23.6	14.5	8.0	6.0	14.1
	*X^2^*		3.768	3.865	0.614	3.172	0.291
	P		0.052	0.049	0.433	0.075	0.590
Rurality	Rural	314	19.1	9.6	6.4	4.0	9.2
	Urban	689	22.2	13.9	7.8	9.0	15.5
	*X^2^*		1.238	3.766	0.680	7.438	7.290
	P		0.266	0.052	0.410	0.006	0.007
Ethnicity	Other	69	20.3	10.1	8.7	12.9	15.9
	White British	934	21.3	12.7	7.3	7.0	13.4
	*X^2^*		0.040	0.394	0.188	2.889	0.359
	P		0.842	0.530	0.664	0.089	0.549

# *Experienced more than rarely in last 12 months, see methods;*

* *Work excludes those who are neither in employment or students (n = 61)*.

∧ *Higher DOAT (Digital Overuse and Addiction Traits) score is calculated as one standard deviation or more above the sample mean (see methods)*.

Bangor University Healthcare and Medical Sciences Academic Ethics Committee and Public Health Wales Research and Development Office provided ethical approval for this study to be conducted (References: 2018-16286 & 2018PHW0004). Statistical analyses used chi-squared for bivariate comparisons. Cronbach's alpha was used to measure of internal consistency of Likert questions combined into the DOAT scale. Binary and multinomial logistic regression techniques were used to examine the independent association of multiple independent variables with outcomes of interest and region of sampling included in all models to reflect the sampling framework.

For all models, socio-demographic variables are included as covariates primarily to correct for differences that might arise from demographic variations across other variables of interest. However, relationships between outcomes of interest and socio-demographic variables are reported unadjusted as percentages and adjusted through logistic regression modelling and discussed where appropriate. Other variables tested against outcomes of interest were considered independent variables. All statistics were undertaken in SPSS version 25 and significance level was set as *p* < 0.05. The Market Research Society Code of Conduct and the Declaration of Helsinki were adhered to by all interviewers. The study fully complies with Strengthening the Reporting of Observational Studies in Epidemiology (STROBE) guidelines (46). Verbal informed consent was obtained from all participants prior to participation.

## Results

### Prevalence and Socio-Demographics of Overuse and Addiction

Overall, just over one in five individuals (21.2%) reported having unsuccessfully tried to cut down on their digital technologies use in the past year. One in eight respondents (12.5%) reported feeling restless when stopped from using digital technologies and just over one in 14 (7.4%) reported a negative impact on home and social life or work (Table 1). Around one in seven (13.6%) individuals had a higher DOAT, and proportions reduced significantly with age (18–29 years: 22.8%; 60+ years: 4.2%). All individual components in the scale also reduced in prevalence as age increased (Table 1). There were no significant relationships between the individual components of DOAT and deprivation. However, individuals in the poorest quintile were more likely to have a higher DOAT (20.9%) compared with levels between 10.3% and 14.1% in other quintiles. Feeling restless when stopped from using digital technologies was related to being female, but no other components of the DOAT scale or having a higher DOAT was associated with gender. Those with an urban residence were also more likely to have a higher DOAT and report a negative impact of too much digital use on jobs/studies. Ethnicity was not significantly related to any component of DOAT or the overall scale (Table 1).

### Relationships Between DOAT and Mental Well-Being, Online Activity, and Other Addictive Behaviours

Across the whole sample, 13.2% of individuals were categorised as having LMWB (see Methods). Individually reporting any component of the DOAT scale or having a higher DOAT overall were positively related to LMWB with membership of the higher DOAT category more than doubling proportions reporting LMWB (Table 2). When corrected for other factors (Logistic Regression, Table 3), LMWB remained independently associated only with restlessness when stopped from using, negative impacts of too much use on job/studies and with a higher overall DOAT. Across levels of social media use, there were also strong positive relationships with each of the DOAT components as well as with having a higher overall DOAT. High social media use remained significantly associated with failure to cut down, restlessness when stopped using as well as having a higher overall DOAT after correction for other factors (Table 3). In bivariate analyse, frequency of internet access was positively related to a higher DOAT as well as higher levels of all DOAT components except reporting negative impacts from overuse on home and social life. In logistic regression, high internet access frequency remained significantly related to a higher DOAT, failure to cut down and having a negative impact on job/studies.

**Table 2:** Digital Overuse and Addiction Traits (DOAT) by mental well-being, online activity, current tobacco smoker, and binge drinker.

			**% Categorised “yes”^#^**				**% Higher DOAT^∧^**
		***n***	**Failed to cut down**	**Restless when stopped using**	**Negative impact on home/social**	Negative impact on work^*^	
Lower mental well-being^£^	No	871	20.0	11.0	6.7	6.6	11.9
	Yes	132	29.5	22.7	12.1	12.8	24.2
	*X^2^*		6.274	14.299	5.005	6.040	14.801
	P		0.012	<0.001	0.025	0.014	<0.001
Social media use tertiles^‡^	Low	359	10.0	5.3	3.6	3.7	5.8
	Moderate	330	21.5	12.4	7.3	8.3	12.4
	High	314	33.8	21.0	11.8	10.5	23.6
	*X^2^*		56.413	37.723	16.338	10.827	45.399
	P		<0.001	<0.001	<0.001	0.004	<0.001
Frequency of internet access tertiles	Low	465	15.9	9.7	6.7	4.3	9.2
	Moderate	335	22.4	1.7	6.6	8.5	13.7
	High	203	31.5	17.2	10.3	12.5	23.2
	*X^2^*		20.993	7.986	3.281	14.167	23.325
	P		<0.001	0.018	0.194	<0.001	<0.001
Current smoker	No	840	20.1	11.8	6.4	7.4	12.6
	Yes	163	27.0	16.6	12.3	7.8	18.4
	*X^2^*		3.857	2.838	6.816	0.045	3.899
	P		0.049	0.092	0.009	0.832	0.048
Binge drinker	No	844	20.5	11.6	6.9	7.0	12.7
	Yes	159	25.2	17.6	10.1	9.9	18.2
	*X^2^*		1.737	4.383	1.993	1.565	3.530
	P		0.188	0.036	0.158	0.211	0.060

# *Experienced more than rarely in last 12 months, see methods; DOAT, Digital Overuse and Addiction Traits score*.

* *Work excludes those who are neither in employment or students (n = 61)*.

∧ *Higher DOAT (Digital Overuse and Addiction Traits) score is calculated as one standard deviation or more above the sample mean (see methods)*.

£ *Mental well-being is calculated using the Short Warwick-Edinburgh Mental Well-being Scale (SWEMWBS). Lower mental well-being was calculated as below one standard deviation from the sample mean*.

‡ *Social media use was calculated as tertiles of a cumulative score (see methods)*.

**Table 3:** Logistic regression analysis of factors associated with having a high Digital Overuse and Addiction Traits (DOAT) score.

		**Failed to cut down**		**Restless when stopped using**		**Negative impact on home/social**		Negative impact on work^*^		**Higher DOAT^∧^**	
		***P***	**AOR (95% CI)**	***P***	**AOR (95% CI)**	***P***	**AOR (95% CI)**	***P***	**AOR (95% CI)**	***P***	**AOR (95% CI)**
Lower mental well-being (Ref = No)	Yes	0.198	1.35 (0.86–2.12)	0.003	2.14 (1.29–3.57)	0.403	1.31 (0.69–2.50)	0.014	2.25 (1.18–4.31)	0.003	2.15 (1.30–3.55)
Social media use Tertiles	(Ref) Low	0.013		0.013		0.712		0.667		0.032	
	Moderate	0.192	1.39 (0.85–2.26)	0.028	2.04 (1.08–3.84)	0.919	1.04 (0.48–2.27)	0.422	1.38 (0.63–3.04)	0.373	1.32 (0.72–2.45)
	High	0.005	2.11 (1.25–3.56)	0.003	2.80 (1.41–5.56)	0.517	1.31 (0.58–2.93)	0.774	1.13 (0.48–2.66)	0.018	2.17 (1.14–4.14)
Internet access frequency Tertiles	(Ref) Low	0.013		0.223		0.246		0.077		0.002	
	Moderate	0.499	1.15 (0.77–1.72)	0.785	1.07 (0.66–1.75)	0.318	0.73 (0.39–1.36)	0.282	1.45 (0.74–2.83)	0.513	1.18 (0.72–1.94)
	High	0.005	1.91 (1.21–3.01)	0.109	1.57 (0.90–2.74)	0.491	1.27 (0.64–2.51)	0.025	2.29 (1.11–4.73)	0.001	2.44 (1.42–4.17)
Current smoker (Ref = No)	Yes	0.526	1.15 (0.74–1.78)	0.712	1.11 (0.65–1.88)	0.251	1.43 (0.78–2.65)	0.817	1.09 (0.53–2.23)	0.512	1.19 (0.71–1.98)
Binge drinker (Ref = No)	Yes	0.761	1.07 (0.70–1.64)	0.220	1.36 (0.83–2.24)	0.574	1.20 (0.64–2.22)	0.667	1.15 (0.61–2.18)	0.397	1.24 (0.76–2.03)
Age (years)	18–29	<0.001	4.15 (2.07–8.34)	0.076	2.02 (0.93–4.40)	0.001	7.80 (2.27–26.86)	0.018	4.08 (1.28–13.02)	0.012	2.94 (1.27–6.85)
	30–39	<0.001	4.22 (2.18–8.16)	0.548	1.26 (0.60–2.66)	<0.001	9.27 (2.83–30.41)	0.086	2.70 (0.87–8.37)	0.007	2.97 (1.34–6.60)
	40–49	<0.001	4.38 (2.30–8.33)	0.619	1.21 (0.57–2.56)	0.003	5.98 (1.83–19.54)	0.040	3.18 (1.05–9.58)	0.018	2.62 (118–5.79)
	50–59	0.085	1.84 (0.92–3.67)	0.678	0.84 (0.38–1.89)	0.937	1.07 (0.23–4.99)	0.992	0.99 (0.27–3.67)	0.706	1.19 (0.49–2.89)
	60+ (Ref)	<0.001		0.148		<0.001		0.035		0.018	
Gender (Ref = Male)	Female	0.054	1.40 (0.99–1.96)	0.080	1.45 (0.96–2.19)	0.575	1.16 (0.69–1.96)	0.080	0.62 (0.37–1.06)	0.522	1.14 (0.76–1.70)
Deprivation	(Ref) Poor 1	0.877		0.431		0.469		0.673		0.119	
	2	0.779	0.93 (0.55–1.56)	0.088	0.57 (0.30–1.09)	0.304	0.66 (0.29–1.47)	0.431	0.70 (0.29–1.69)	0.020	0.47 (0.25–0.89)
	3	0.363	0.77 (0.44–1.35)	0.297	0.71 (0.37–1.36)	0.380	0.68 (0.29–1.60)	0.841	1.09 (0.47–2.54)	0.110	0.59 (0.31–1.13)
	4	0.808	0.93 (0.54–1.62)	0.860	0.94 (0.50–1.79)	0.610	1.23 (0.56–2.68)	0.529	1.31 (0.57–2.99)	0.771	0.91 (0.49–1.69)
	Affluent 5	0.933	1.02 (0.59–1.77)	0.653	0.87 (0.46–1.63)	0.797	1.11 (0.50–2.48)	0.582	1.26 (0.55–2.87)	0.274	0.71 (0.38–1.32)
Rurality (Ref = Rural)	Urban	0.884	0.97 (0.64–1.47)	0.978	1.01 (0.60–1.69)	0.577	0.84 (0.44–1.58)	0.279	1.53 (0.71–3.29)	0.619	1.14 (0.67–1.94)
Health Board Sampling Strata^£^		0.019		0.297		0.395		0.669		0.095	

*DOAT, Digital Overuse and Addiction Traits score; AOR, Adjusted odds ratio; 95% CI, 95% confidence interval; Ref, Reference category*.

* *Work excludes those who are neither in employment or students (n = 61)*.

∧ *Higher DOAT (Digital Overuse and Addiction Traits) score is calculated as one standard deviation or more above the sample mean (see methods)*.

£ *Overall P-values for the variable health board sampling strata are shown but P-values for each individual sampling area are not included in order to limit table size*.

In bivariate analyses, being a current smoker was related to higher failure to cut down use, negative impacts from overuse on home and social life, and having a higher overall DOAT. Only restlessness when stopped from using devices was associated with alcohol bingeing (Table 2). However, no associations with smoking or drinking remained significant after correcting for other factors (Table 3). Logistic regression analyses identified no relationships between a higher DOAT or DOAT components with deprivation or gender (Table 3). However, all DOAT component measures except restlessness when stopped from using digital technology were independently associated with age, with those aged 50 years or more least likely to report digital overuse or addiction traits (Table 3). For all age groups under 50 years, likelihood of reporting a negative impact on home and social life from overuse was more than five times higher than in those aged 60 years or over.

### Online Well-Being Outcomes and Their Relationships With DOAT and Mental Well-Being Status

Individuals were asked to rate how their experience of using the internet for any reason related to health (physical or mental) impacted their well-being across three measures (self-esteem, feeling isolated, anxiety about health). Most individuals reported no impact from their internet use (self-esteem: 88.0%; feeling isolated: 88.6%; anxiety about health: 84.5%). However, for each well-being measure, individuals with a higher DOAT score were significantly more likely to report an impact, either positive or negative (Table 4). Thus, for self-esteem, individuals with a higher DOAT were 2.6 times more likely to report self-esteem being made worse and nearly 4 times more likely to report it being made better (cv. those without a higher DOAT, who were statistically more likely to report no impact of internet use on their self-esteem; Table 4). Similar significant relationships were seen between a higher DOAT and both feelings of isolation and anxiety about their health, although the odds of internet use making things worse were higher than making them better in these cases.

**Table 4:** Multinomial logistic regression analysis of factors related to mental well-being outcomes when using the internet for health-related issues.

		**Self-esteem (Ref = No change;** ***n*** **= 883)**					**Feeling isolated (Ref = No change;** ***n*** **= 889)**					**Anxiety about health (Ref = No change;** ***n*** **= 848)**				
			**Worse (*****n*** **= 30)**		**Better (*****n*** **= 90)**			**Worse (*****n*** **= 24)**		**Better (*****n*** **= 90)**			**Worse (*****n*** **= 24)**		**Better (*****n*** **= 90)**	
		Pm*	***P***	**AOR (95% CI)**	***P***	**AOR (95% CI)**	Pm*	***P***	**AOR (95% CI)**	***P***	**AOR (95% CI)**	Pm*	***P***	**AOR (95% CI)**	***P***	**AOR (95% CI)**
DOAT score (Ref = Not higher)	Higher	<0.001	0.031	2.64 (1.09–6.40)	<0.001	3.86 (2.26–6.60)	<0.001	0.001	5.60 (2.11–14.91)	0.005	2.22 (1.27–3.86)	<0.001	<0.001	4.80 (2.34–9.86)	<0.001	2.80 (1.68–4.67)
Lower mental well-being (Ref = No)	Yes	0.025	0.009	3.48 (1.37–8.81)	0.185	1.54 (0.81–2.90)	0.002	0.002	4.76 (1.77–12.82)	0.040	1.88 (1.03–3.45)	0.868	0.628	1.23 (0.53–2.85)	0.766	1.10 (0.60–2.00)
Social media use tertiles (Ref = Low)	Moderate	0.062	0.326	0.46 (0.10–2.15)	0.250	1.54 (0.74–3.20)	0.049	0.025	0.14 (0.02–0.78)	0.479	1.27 (0.65–2.47)	0.122	0.448	0.66 (0.22–1.94)	0.246	1.44 (0.78–2.67)
	High		0.822	0.84 (0.19–3.67)	0.010	2.79 (1.27–6.10)		0.624	0.71 (0.18–2.76)	0.174	1.66 (0.80–3.45)		0.540	1.40 (0.48–4.15)	0.038	2.05 (1.04–4.02)
Internet access frequency tertiles (Ref = Low)	Moderate	0.194	0.217	1.98 (0.67–5.86)	0.652	1.14 (0.64–2.05)	0.688	0.634	0.75 (0.22–2.49)	0.598	1.16 (0.67–2.00)	0.078	0.162	1.82 (0.79–4.22)	0.731	1.10 (0.65–1.86)
	High		0.023	3.97 (1.21–13.09)	0.289	1.43 (0.74–2.76)		0.438	1.65 (0.47–5.82)	0.812	0.92 (0.48–1.79)		0.140	2.09 (0.79–5.57)	0.020	1.95 (1.11–3.42)
Current smoker (Ref = No)	Yes	0.687	0.486	1.48 (0.49–4.45)	0.647	0.86 (0.44–1.66)	0.429	0.317	0.50 (0.13–1.95)	0.411	0.76 (0.39–1.47)	0.714	0.862	1.08 (0.45–2.60)	0.445	0.79 (0.43–1.45)
Binge drinks weekly or more (Ref = No)	Yes	0.181	0.629	1.26 (0.49–3.27)	0.101	0.55 (0.27–1.12)	0.695	0.998	1.00 (0.32–3.11)	0.406	0.76 (0.40–1.45)	0.448	0.333	0.64 (026–1.59)	0.379	0.76 (0.42–1.39)
Age (years; Ref = 60+)	18–29	0.019	0.021	9.20 (1.39–61.03)	0.282	1.68 (0.65–4.32)	0.101	0.049	6.40 (1.01–40.64)	0.320	1.62 (0.64–4.18)	0.168	0.029	6.64 (1.21–36.40)	0.679	1.19 (0.53–2.68)
	30–39		0.122	4.32 (0.68–27.62)	0.167	1.87 (0.77–4.56)		0.145	3.90 (0.63–24.30)	0.008	3.10 (1.34–7.19)		0.044	5.60 (1.04–30.01)	0.683	1.17 (0.55–2.48)
	40–49		0.656	0.56 (0.04–7.11)	0.261	1.65 (0.69–3.95)		0.289	2.71 (0.43–17.04)	0.143	1.91 (0.80–4.52)		0.020	6.76 (1.35–33.93)	0.418	1.35 (0.65–2.79)
	50–59		0.740	0.66 (0.06)	0.689	1.21 (0.48–3.04)		0.530	1.88 (0.26–13.55)	0.241	1.68 (0.71–4.00)		0.799	1.30 (0.17–9.76)	0.841	1.08 (0.51–2.27)
Gender (Ref = Male)	Female	0.103	0.041	2.56 (1.04–6.29)	0.818	1.06 (0.66–1.71)	0.518	0.403	0.67 (0.27–1.70)	0.401	0.82 (0.52–1.30)	0.021	0.443	1.31 (0.66–2.61)	0.007	1.83 (1.18–2.83)
Deprivation (Ref = Poorest 1)	2	0.108	0.115	3.11 (0.76–12.78)	0.795	1.12 (0.49–2.57)	0.555	0.324	2.08 (0.49–8.93)	0.588	0.80 (0.35–1.81)	0.965	0.892	1.08 (0.37–3.11)	0.918	1.04 (0.52–2.06)
	3		0.403	1.89 (0.43–8.41)	0.355	1.48 (0.64–3.41)		0.260	2.52 (0.51–12.54)	0.319	1.48 (0.69–3.18)		0.328	0.54 (0.16–1.86)	0.690	0.86 (0.42–1.79)
	4		0.240	2.42 (0.55–10.63)	0.006	2.95 (1.35–6.44)		0.109	3.58 (0.75–17.05)	0.253	1.56 (0.73–3.34)		0.763	0.85 (0.28–2.53)	0.778	1.11 (0.55–2.24)
	5		0.425	1.82 (0.42–7.97)	0.488	1.35 (0.58–3.16)		0.216	2.91 (0.54–15.75)	0.395	1.39 (0.65–2.98)		0.867	1.09 (0.39–3.08)	0.853	0.93 (0.46–1.92)
Rurality (Ref = Rural)	Urban	0.563	0.301	0.56 (0.19–1.69)	0.793	1.08 (0.60–1.98)	0.555	0.974	1.02 (0.28–3.70)	0.276	0.73 (0.42–1.28)	0.084	0.235	0.61 (0.27–1.38)	0.084	1.64 (0.94–2.87)
Health Board Sampling Strata^∧^		0.024					0.042					0.096				

*DOAT, Digital Overuse and Addiction Traits score; AOR, Adjusted odds ratio; 95% CI, 95% confidence interval; Ref, Reference category; Pm, Overall P value for the independent variable*.

∧ *Overall P values for the variable health board sampling strata are shown but P-values for each individual sampling area are not included in order to limit table size*.

For LMWB, a similar pattern was identified of internet use being associated with improved feelings of isolation or with making them worse; the odds of making it worse were also greater than those for making it better (AOR: 4.76 vs. 1.88). LMWB was associated with internet use for health reasons reducing self-esteem but not improving it and showed no significant relationships with anxiety about health. There was an association between levels of social media use (a reduced risk of feelings of isolation with moderate compared to low levels of use) but otherwise levels of social media use and internet access frequency were not significantly related to well-being outcomes measured. Individuals aged 18–29 years were most likely to report use of the internet for health reasons having made their self-esteem, isolation, and anxiety worse (6–9 times more than those aged 60+ years). The only age difference significantly associated with internet use resulting in an improvement was in feelings of isolation amongst individuals aged 30–39 years. Internet use for health was more likely to be reported as related to reduced anxiety about health and to worsening self-esteem in women. Deprivation and rurality were not significantly related to any reported negative or positive changes in measures of well-being with their experience of internet use.

## Discussion

Whilst internationally uptake of digital technologies has been initially greater in younger age groups, year on year increases in internet use and associated technology ownership (e.g., smartphones) are a feature across all age groups (4, 28, 47). Political, as well as commercial, activities continue to encourage all sectors of society to be digitally connected. As well as social connectivity [93% of 16–24 year olds and 58% of 55–64 year olds in the UK have a social media account (47)], key health information and services are sometimes only accessible online and ensuring wider connectivity is critical for avoiding a digital divide with subsequent impacts on health inequalities (48). However, as the use of digital technology becomes unavoidable, we need to consider the extent and consequences of overuse along with addiction. In this national survey of Wales, 85.0% of individuals had internet access at their place of residence or used the internet at least weekly elsewhere (19). Of these, more than one in five had tried to cut down their digital technology use in the past year but failed to do so. Results are consistent with surveys elsewhere which have found younger individuals report more digital overuse (13).

Results here identified trying but failing to cut down use during the last year affected 31.7% in those aged 18–29 years but was still reported by 28.3% in those aged 40–49 years with larger falls in prevalence only seen in those aged 50 years or over (Table 1). Restlessness when stopped from using (12.5%) and negative impacts on home/social (7.4%) and work environments (7.4%) were less common but also showed higher prevalence in those aged under 50 years (Table 1). Few recent studies have examined relationships between digital overuse and deprivation, yet the relationship with higher education level (a component of deprivation) has been found to be significantly associated with both greater risk and protection from perceived digital overuse (13). Here, no significant relationship was found between being in a higher category for DOAT (or the individual constructs) and deprivation (Table 3) and with the exception of age, most other demographic factors were not strongly related to either individual components of the DOAT scale or having an overall higher DOAT (Table 3). Thus, at least in those under 50 years, results suggest DOATs are relatively widespread features across genders, deprivation quintiles and both urban and rural settings (Table 3).

Evidence elsewhere suggests that while a moderate level of technology use does not lead to negative impacts on mental well-being (15), high levels carry a risk. Thus, in a UK study of adolescents, social media use was “perceived as a threat to mental well-being” by the participants and considered addictive, promoting mood and anxiety disorders (49). Digital technology use for entertainment purposes was found to be the most significant predictor of LMWB rather than general habitual use or use for communication purposes (21). Our study also identified a higher DOAT to be associated with high frequencies of internet access and social media use but not with moderate use (Table 3).

Less consideration has been given to whether those with LMWB may be more vulnerable to overuse and addiction. This study relies on associations between variables collected at a single point in time and consequently causality cannot be established. However, results here indicate that, independent of levels of internet and social media activity, those with poorer mental well-being may be at greater risk of overuse (Table 3). Thus, restlessness when stopped from using digital technology and negative impacts on job/studies both increased with LMWB; with LMWB associated with more than a doubling in likelihood of a higher DOAT (Tables 1, 3). A nationally representative survey in Switzerland also found higher PDO was associated with lower well-being (9). Moreover, research focusing on social media use and mental health outcomes such as depression also find significant associations between greater use and higher levels of depression; although negative impacts are generally small (49, 50). Investment, especially by the public sector, to recruit people to the use of digital technologies and increase their engagement should consider its impact on those with LMWB or other vulnerabilities to DOAT and how it can also protect them from other commercial sectors seeking to increase their digital engagement.

Whether LMWB is a result of DOAT, a cause of DOAT or that DOAT and LMWB are related to each other through other factors requires further study. However, the DOAT scale employed here encompasses measures of failure to control use (e.g., failed attempts to cut down), and a recent systematic review of social networking sites and depression and anxiety (51) suggested that, as technology is a part of everyday life, finding ways to help people control their use rather than abstain from using digital technology would be more apt. Given that many daily functions are now centred on technology, it has properties that can naturally lead to dependency (26). Critically however, our study also examined the self-reported impact of using the internet for health in general on a number of well-being outcomes. Most individuals reported no change in measures of self-esteem, feelings of isolation and anxiety about their health as a result of such use (Table 4). However, those aged 18–29 years were significantly more likely to report worsening of self-esteem as a result of their use, even after controlling for levels of internet and social media activity. This relationship between lower self-esteem and increased online activity is consistent with studies of youth elsewhere (50); although one review highlighted that self-esteem can be improved if the internet activity yields positive online interactions (51). Moreover, individuals with a higher DOAT were substantially more likely to report either benefit or harm to self-esteem, feeling isolated or anxiety about health as a result of their internet use. In the case of feeling isolated and anxiety the risk of increased harm appears greater than the risk of improvement (Table 4).

Self-reported changes in health anxiety resulting from internet use for health were not related to LMWB unlike changes in self-esteem and isolation (Table 4). However, likelihood of improved feelings of isolation from internet use associated with LMWB were less likely (AOR = 1.88) than worsened feelings of isolation (AOR = 4.76; Table 4). Individuals with LMWB were over three times more likely to report internet use making their self-esteem worse but LMWB made no significant difference to odds of improved self-esteem. Far from a unidirectional relationship, our results suggest that individuals with a higher DOAT and, to a lesser extent, LMWB are more likely to experience benefits or harms through the use of the internet for health purposes. Further study is needed to identify the conditions which maximise the probability of benefits and protect against risks of harm. Findings also suggest caution should be applied when extrapolating from one person, or a small groups of individuals, with a higher DOAT or LMWB to how digital technology use will affect others with similar characteristics. Moreover, our results do not provide data on whether individuals with a higher DOAT or LMWB may switch between finding internet use for health beneficial and harmful or how frequently this might occur. Longitudinal data is required to understand whether relationships with digital technology fluctuate with age and over time. However, collecting such data comes with design challenges due to how quickly digital technology develops [see (52) for overview].

### Limitations

This study was designed as an initial exploration of the prevalence and socio-demographic distribution of digital overuse and addiction measures along with their association with measures of potential vulnerability (e.g., LMWB) and outcomes from online activity. It did not seek to explore a specific theoretical model although the findings may allow further speculation on the composition of such models which can subsequently be tested.

Consistent with studies elsewhere (43, 44), we utilised one standard deviation below the sample mean as a cut off for lower mental well-being. To have a consistent analytical approach, we adapted this statistical approach to define a higher DOAT. This dichotomy allowed the identification of statistically significant relationships, but further work is required to establish how relationships with outcomes of interest are associated with increments in the score across the scale. The cross-sectional study design limited any inferences about causality and the study was only able to establish associations between variables measured. Moreover, other potential variables measuring overuse and addiction and duration of negative impacts on home, social and work lives were not included in this initial survey. Longitudinal studies are required to establish how overuse, addiction and their related impacts on health and well-being develop and may change over time. Although we found no relationships between DOAT and smoking or binge drinking, larger more sensitive scales than those used here for measuring both DOAT and consumption of either substances are available and might provide better insights.

Many of the measures recorded were self-assessed and whilst questions were selected from established scales wherever possible, we cannot account for any subjective bias that may have affected responses across the sample or in specific demographics. Finally, although compliance was 75.4% results could not identify if the patterns of behaviour amongst refusals were consistent with respondents or if their inclusion would have changed overall findings.

### Conclusions

The pace of change in digital technology and its central position in health and all other aspects of life, present a series of challenges for public health research. Moreover, the recent global COVID-19 pandemic has accelerated the movement of health services onto digital platforms (53), as well as increasing the use of social media and video communications (7). The long-term consequences of migrating many aspects of health, social and other areas of life on to digital platforms will not be apparent potentially for decades, during which digital interfaces and services will inevitably change again. However, as digital platforms exert their dominance over more traditional forms of information and social provision, it is critical that people are capable of constraining their activities; especially when they recognise they may be adversely impacting their health. Our results suggest that during a 12-month period over a fifth of individuals tried and failed to reduce internet related activity. Further, substantial proportions of individuals already report negative impacts on home, social and working lives, and pressures to use digital services are likely to increase as more aspects of life go online, such as healthcare (53). To date, guidance, support, and regulations have largely considered adolescents and young children (54), but findings here suggest overuse is a feature of all age groups and shows little decline in prevalence at least until the age of 50 years. With so many regarding their use as too high and unable to reduce it, there is an urgent need for more policies and interventions that help individuals of all ages to find healthy levels of digital technology use. Whilst voluntary measures are already common, research is urgently required to establish if and where statutory requirements are needed, and which would be most effective. Importantly, results here suggest individuals with LMWB have increased vulnerability to overuse and the negative impacts of multiple aspects of life. Moreover, across parameters of self-reported self-esteem, isolation, and anxiety, those that score highly on overuse and LMWB measures are more likely to report both harms and benefits from their digital activities. Vulnerabilities, such as LMWB, need to be reflected in the development of guidance and policy. However, as online activities continue to increase, maximising benefits to public health depends in part on policy and guidance protecting against harms associated with overuse and understanding why, even at similar levels of use, the mental health of some individuals is improved whilst that of others may deteriorate.

## Data Availability Statement

The raw data supporting the conclusions of this article will be made available by the authors, without undue reservation.

## Ethics Statement

The studies involving human participants were reviewed and approved by Bangor University Healthcare and Medical Sciences Academic Ethics Committee and Public Health Wales Research and Development Office provided ethical approval for this study to be conducted (References: 2018-16286 & 2018PHW0004). Verbal informed consent was obtained and recorded from all participants prior to participation.

## Author Contributions

ARD conceived the wider survey. ARD, CAS, and MAB designed the survey questionnaire. ARD and CAS undertook management of data collection. MAB conceived this research topic and undertook analyses. MAB and CAS wrote the initial draught of this manuscript, supported by critical reviewing of analysis, and extensive editing by KH. ARD contributed to manuscript editing. All authors contributed to the article and approved the final version.

## Conflict of Interest

The authors declare that the research was conducted in the absence of any commercial or financial relationships that could be construed as a potential conflict of interest.
