# Supplementary material for: Digital Overuse and Addictive Traits and Their Relationship With Mental Well-Being and Socio-Demographic Factors: A National Population Survey for Wales
Source: Front Public Health. 2021 Jun 16;9:585715. doi: 10.3389/fpubh.2021.585715 (PMC8242162; doi:10.3389/fpubh.2021.585715)
Supplement: Supplementary file 1 [file Table_1.DOC]

**Appendix Table 1. Questions and response option for key dependent and independent variables**

| **Aspect** | **Questions** | **Response options** |
| --- | --- | --- |
| Social media use | *How often do you go on these types of social media websites or apps?* | Several times a day, Daily, Weekly, Less than weekly, Never |
|  | Asked individually for Twitter, Facebook, LinkedIn, Instagram, YouTube, WhatsApp, Snapchat |  |
| Internet access technology | *How often do you access the internet, either at home, work or elsewhere on a* | Several times a day, Daily, Weekly, Less than weekly, Never |
|  | Asked individually for Mobile/Smartphone, Desktop/laptop, Tablet |  |
| Digital Overuse and Addictive Traits (DOAT) questions | *Thinking about the technology you use (which may include phone, computer, tablet) how often in the past year have you* | Never, Rarely, Sometimes, Often, Very Often, Don't know, N/A. |
|  | Tried to cut down on your use of your technology without success |  |
|  | Become restless or troubled if you were not allowed to use technology |  |
|  | Used your technology so much that it has had a negative impact on your home or social life |  |
|  | Used your technology so much that it has had a negative impact on your job/studies |  |
| Mental well-being | *Below are some statements about feelings and thoughts. Please tick the box that best describes your experience of each over the last 2 weeks.* | None of the time, Rarely, Some of the time, Often, All of the time |
|  | I’ve been feeling optimistic about the future |  |
|  | I've been feeling useful |  |
|  | I’ve been feeling relaxed |  |
|  | I’ve been dealing with problems well |  |
|  | I’ve been thinking clearly |  |
|  | I’ve been feeling close to other people |  |
|  | I’ve been able to make up my own mind about things |  |
| Other potentially addictive behaviours | *In terms of tobacco, which of these best describes you* | Never smoked, Smoke daily, Smoke occasionally but not daily, Used to smoke but not at all now |
|  | *In the last year how often have you had 6 or more alcoholic drinks in a single occasion [Binge drinking defined as six or more drinks in one occasion]** | Never, Less than monthly, Monthly, Weekly, Daily or almost daily, I don't drink alcohol at all |
| Changes in health and well-being | *In your opinion has you experience of using the internet and technology to support your health made the following things better or worse?* | Better, Worse, No difference/Don't know |
|  | Your levels of anxiety about your health |  |
|  | Your self-esteem |  |
|  | Feeling isolated |  |
